# Construction of DNA Biosensors for Mercury (II) Ion Detection Based on Enzyme-Driven Signal Amplification Strategy

**DOI:** 10.3390/biom11030399

**Published:** 2021-03-08

**Authors:** Shuchang Wang

**Affiliations:** School of Life Sciences, East China Normal University, Shanghai 200241, China; shuchang5131@sina.com or 10181900234@stu.ecnu.edu.cn

**Keywords:** mercury (II) ion, DNA biosensor, enzyme-driven signal amplification, nuclease, DNAzymes

## Abstract

Mercury ion (Hg^2+^) is a well-known toxic heavy metal ion. It is harmful for human health even at low concentrations in the environment. Therefore, it is very important to measure the level of Hg^2+^. Many methods, reviewed in several papers, have been established on DNA biosensors for detecting Hg^2+^. However, few reviews on the strategy of enzyme-driven signal amplification have been reported. In this paper, we reviewed this topic by dividing the enzymes into nucleases and DNAzymes according to their chemical nature. Initially, we introduce the nucleases including Exo III, Exo I, Nickase, DSN, and DNase I. In this section, the Exo III-driven signal amplification strategy was described in detail. Because Hg^2+^ can help ssDNA fold into dsDNA by T-Hg-T, and the substrate of Exo III is dsDNA, Exo III can be used to design Hg^2+^ biosensor very flexibly. Then, the DNAzyme-assisted signal amplification strategies were reviewed in three categories, including UO_2_^2+^-specific DNAzymes, Cu^2+^-specific DNAzymes and Mg^2+^-specific DNAzymes. In this section, the Mg^2+^-specific DNAzyme was introduced in detail, because this DNAzyme has highly catalytic activity, and Mg^2+^ is very common ion which is not harmful to the environment. Finally, the challenges and future perspectives were discussed.

## 1. Introduction

Mercury ion (Hg^2+^) is a well-known toxic heavy metal ion. It comes from both natural sources (such as oceanic emissions and volcanic eruptions) [1,2] and anthropogenic sources (such as the mining industry, combustion of fossil fuels and solid waste) [3,4,5]. Since Hg^2+^ has high affinity to thiol groups to form Hg-S chemical bonds, it can cause the inactivation of some enzymes and proteins thus leading to some illnesses such as loss of hearing, speech, vision or motor and cognitive disorders [6,7,8]. Meanwhile, Hg^2+^ can also accumulate in human body through the food chain, so it is harmful for human health even at low concentrations in the environment [9]. Additionally, Hg^2+^ can be converted to methyl mercury under the action of organisms such as living bacteria [8]. Methyl mercury is a very toxic neurotoxin which can cause permanent brain damage brain. The World Health Organization (WHO) and the USA Environmental Protection Agency (EPA) have regulated the maximum contaminant level for Hg^2+^ to be below 10 nM in drinking water. Therefore, it is very important to measure the level of Hg^2+^.

So far, there have been many methods to detect Hg^2+^ based on instruments (such as atomic absorption spectroscopy) [10,11,12], small organic molecules [13,14,15], proteins [16], DNA [17,18,19] and so on. Among these methods, DNA (oligonucleotide) biosensors have attracted much attention because DNA is stable, cheap, and easy to obtain as well as to modify. In 2004, Ono and Togashi first discovered that Hg^2+^ could specifically bind to thymine (T) to form the T-Hg-T mismatch [20]. Then, they designed the first DNA biosensor to detect Hg^2+^ by modifying quencher and fluorophore at the two ends of the thymine-rich DNA. In fact, the binding constant of T-Hg-T is higher than Watson-Crick pair (T-A), so a T-Hg-T mismatch can easily replace a T-A pair [21,22]. According to this phenomenon, Lu group developed a new biosensor for detecting Hg^2+^ in 2008 [23]. Since then, many DNA biosensors have been designed and developed based on T-Hg-T mismatch [24,25,26]. Since the maximum residue limits (MRL) of Hg^2+^ is as low as only 10 nM, many signal amplification strategies have also been developed concomitantly to improve the sensitivity of the Hg^2+^ DNA biosensors [27,28,29,30,31], such as those based on nanomaterials [30,32] and enzyme-driven signal amplification. Subsequently, because many enzymes are commercial and the quality between batches can be easily controlled, the enzyme-driven signal amplification has become a convenient approach for mercury sensing. Until now, several reviews have been published on DNA biosensors for the detection of Hg^2+^ [33,34,35]. However, few reviews on the strategy of enzyme-driven signal amplification have been reported. In this paper, we reviewed this topic by dividing the enzymes into nucleases and DNAzymes according to their chemical nature. For nucleases, exonuclease III (Exo III), exonuclease I (Exo I), nicking endonuclease (Nickase), duplex-specific nuclease (DSN) and deoxyribonuclease I (DNase I) were used as representatives to illustrate. For DNAzymes, they were divided into three categories, UO_2_^2+^-specific DNAzymes, Cu^2+^-specific DNAzymes and Mg^2+^-specific DNAzymes. This review will help readers better understand the application of enzyme-driven signal amplification strategy in the construction of Hg^2+^ DNA biosensors.

## 2. Nucleases

**Figure 1:**
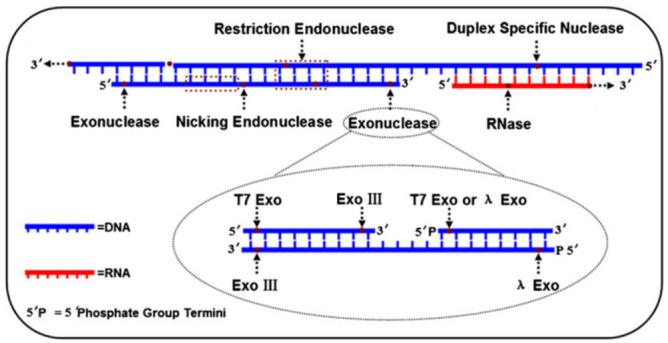
A scheme of different nucleases with different functions. Reprinted with permission from ref. [36]. Copyright 2015 Springer Nature.

Nucleases are a class of enzymes that cleave nucleic acids. Their chemical nature is proteinic. They usually have the structures or sequences which are specific for the substrate therefore are often used as tool enzymes for genetic engineering. With the development of life science, many different kinds of nucleases have been discovered with different functions (Figure 1, Table 1). Generally, they include restriction endonuclease, duplex specific nuclease, exonuclease, nicking endonuclease, RNase, and so on. In DNA biosensors, we can control the formation or disappearance of the nucleases action site by adding target in the designing of the biosensor. In this process, the enzymes are not consumed. Therefore, they can be used for the signal amplification [36,37,38]. Here, we chose five common nucleases (exonuclease III, exonuclease I, nicking endonuclease, duplex-specific nuclease and deoxyribonuclease I) as representatives to illustrate their applications of enzyme-driven signal amplification strategy in Hg^2+^ DNA biosensors.

### 2.1. Exonuclease III

Exonuclease III (Exo III) is a member of the exonuclease family. It can remove the mononucleotides from 3′-end of double-stranded DNA. Typically, it is a double-stranded specific nuclease. The 3′-end overhangs over four bases of double-stranded DNA or single-stranded DNA can prevent the degradation by Exo III. Since Hg^2+^ can promote the single-stranded rich thymine DNA to form double-stranded structure via T-Hg-T mismatch, Exo III can be used to design DNA biosensors by digesting this double-stranded structure to induce a chain of reactions [39,40,41,42].

#### 2.1.1. Exo III-One Cycle

**Figure 2:**
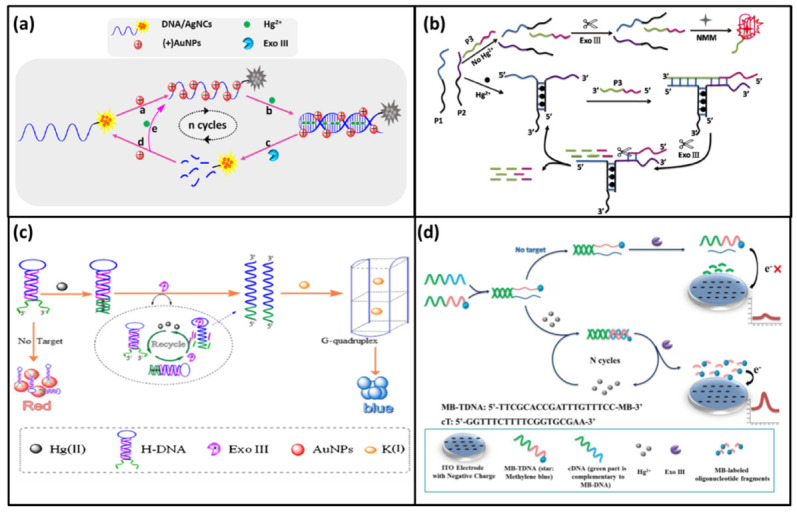
(**a**) A scheme of fluorescence DNA biosensor to detect Hg^2+^ based on Exo III-assisted signal amplification and silver nanoclusters. Reprinted with permission from [43]. Copyright 2019 Springer Nature. (**b**) A scheme of a label-free fluorescence DNA biosensor for detection Hg^2+^ based on Exo III-aided signal amplification and G-quadruplex DNA [26]. (**c**) A scheme of a colorimetric DNA biosensor for the detection of Hg^2+^ using Exo III-assisted signal amplification and AuNPs. Reprinted with permission from [46]. Copyright 2017 Springer Nature. (**d**) A scheme of a homogeneous electrochemical DNA biosensor for Hg^2+^ determination based on Exo III-assisted recycling amplification and indium tin oxide (ITO) electrode. Reprinted with permission from [47]. Copyright 2018 Royal Society of Chemistry.

In 2019, Ma; et al. reported a fluorescence DNA biosensor to detect Hg^2+^ based on Exo III-assisted signal amplification together with silver nanoclusters [43]. This DNA biosensor was a T-rich DNA with silver nanoclusters (DNA-AgNCs). It could bind to positively charged gold nanoparticles (AuNPs) through negatively charged phosphoric acid skeleton. Then, the fluorescence of DNA-AgNCs could be quenched by AuNPs, therefore there was no fluorescence signal in the initial state. In the presence of Hg^2+^, DNA-AgNCs could form a DNA duplex via T-Hg-T mismatch. This structure could be digested into small DNA fragments by Exo III. As a result, the AgNCs were separated from AuNPs and the fluorescence signal was recovered. Meanwhile, Hg^2+^ was also released and could be used in the next cycle (Figure 2a). Therefore, a small amount of Hg^2+^ could cause a huge accumulated increase in fluorescence. Accordingly, the sensitivity of the method was improved with a detection limit as low as 2.3 pM.

In 2018, Ding; et al. developed a label-free fluorescence DNA biosensor for detecting Hg^2+^ based on Exo III-aided signal amplification and G-quadruplex DNA [26]. In this DNA biosensor, three DNA probes (P1, P2, P3) were involved. The 3′-end of P1 and 5′-end of P2 contained poly-T sequences. Meanwhile, the 5′-end of P3 contained a G-quadruplex sequence. In the absence of Hg^2+^, P1, P2, P3 were all in free single stranded DNA state. Thus, they could not be recognized and cut by Exo III. At this status, the G-quadruplex sequence could form a cage-like structure which could bind N-methylmesoporphyrin IX (NMM) to emit fluorescence signal [44,45]. In the presence of Hg^2+^, P1 and P2 could form a T-shaped platform with the help of T-Hg-T mismatch. Then, P3 could bind to this T-shaped platform by base complementation to form a partial double chain structure which could be digested by Exo III. As P1 was degraded, the G-quadruplex sequence was cut and the cage-like structure could not be retained to trap NMM. As a result, the fluorescence signal decreased. Meanwhile, the T-shaped platform was released and entered the next cycle to cause more destruction of G-quadruplex (Figure 2b). At last, the highly sensitive DNA biosensor was realized with a detection limit as low as 1.0 pM.

In 2017, Hong et al. reported a colorimetric DNA biosensor for the detection of Hg^2+^ using Exo III-assisted signal amplification and AuNPs [46]. In this DNA biosensor, a hairpin-shaped DNA probe (H-DNA) with G-quadruplex sequence was designed. In particular, it was rich in T bases at both ends. In the absence of Hg^2+^, the H-DNA could bind with AuNPs and protect them from aggregation in solutions of high ionic strength. In this status, the separate AuNPs showed red color. In the presence of Hg^2+^, the terminal of the H-DNA could form a DNA duplex stem via T-Hg-T mismatch. Then, Exo III could digest this structure from the 3′-terminal of the H-DNA. The digestion of H-DNA led to the release of Hg^2+^ and the 5′-terminal of the H-DNA. The free Hg^2+^ kept on binding the next H-DNA to cause more DNA to be cut. In the presence of K^+^, the free 5′-terminal of the H-DNA could form the G-quadruplex structure which lost the ability to protect AuNPs. Thus, the aggregated AuNPs led to a color change from red to blue in the high ionic solutions. The color change could be easily observed by the naked eye (Figure 2c). With the Exo III-assisted signal amplification strategy, the ultrasensitive colorimetric DNA biosensor was obtained with the detection limit as low as 3.2 pM.

In addition to fluorescence and color methods, electrochemical methods have also been used to detect Hg^2+^. In 2018, Fu; et al. reported a homogeneous electrochemical DNA biosensor for Hg^2+^ determination based on the Exo III-assisted recycling amplification and indium tin oxide (ITO) electrode [47]. In this DNA biosensor, methylene blue (MB) was modified at the 3′-end of the DNA probe to form MB-TDNA. The poly T sequence was also introduced at the 3′-end of MB-TDNA. The partially complementary DNA (cDNA) of MB-TDNA with poly T sequence in its 5′-end was used (Figure 2d). In the absence of Hg^2+^, the 5′-end of MB-TDNA and 3′-end of cDNA could form a corresponding duplex. Subsequently, the cDNA was cut by Exo III, however, MB-TDNA kept intact. Because the phosphate backbone of MB-TDNA had a large negative charge which was repulsed from the negatively charged ITO electrode, the electrochemical signal was low. When Hg^2+^ was added, it helped cDNA and MB-TDNA form a complete double chain structure via T-Hg-T mismatch. This structure could be digested by Exo III. As a result, MB and Hg^2+^ dissociated from the DNA probe. Then free MB moved close to the surface of ITO electrode to produce a significant current signal. Meanwhile, free Hg^2+^ induced a new hybridization and produced more free MB. This homogeneous electrochemical DNA biosensor had a linear range of 1.0 nM–0.5 μM with a detection limit of 0.38 nM.

#### 2.1.2. Exo III-Multiple Cycles

**Figure 3:**
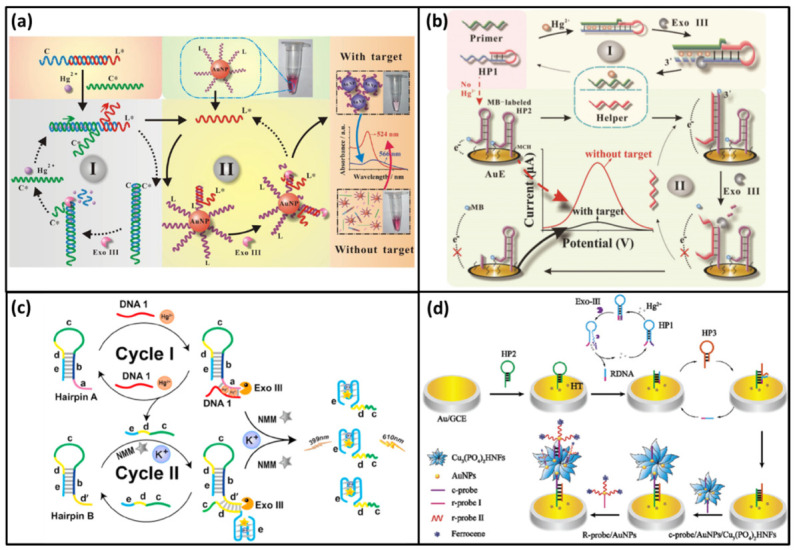
(**a**) A scheme of a colorimetric DNA biosensor for visual detection Hg^2+^ based on Exo III- driven twice cycles signal amplification strategy and AuNPs. Reprinted with permission from ref. [50]. Copyright 2019 Springer Nature. (**b**) A scheme of a ultrasensitive electrochemical for detection Hg^2+^ based on Exo III- assisted double cycles signal amplifi-cation strategy and gold electrodes. Reprinted with permission from ref. [51]. Copyright 2018 Royal Society of Chem-istry. (**c**) A scheme of a fluorescence DNA biosensor for Hg^2+^ detection based on G-quadruplex/NMM as a reporter. Reprinted with permission from ref. [52]. Copyright 2020 Elsevier. (**d**) A scheme of a triple signal amplification strategy for ultrasensitive Hg^2+^ detection. Reprinted with permission from ref. [54]. Copyright 2020 Royal Society of Chemistry.

Except for the single-loop amplification strategy, Exo III can also induce multiple cycle signal amplification to achieve higher sensitivity [48,49]. In 2019, Song et al. developed a colorimetric DNA biosensor for the visual detection of Hg^2+^ based on Exo III-driven double-cycle signal amplification strategy and AuNPs [50]. At the beginning, DNA-C and its partial complementary chain DNA-L* formed a duplex structure. When Hg^2+^ was added, it helped DNA-C* to replace DNA-L* by forming stronger T-Hg-T mismatch. The DNA-C on the DNA-C-C* duplex structure could be degraded by Exo III. Then DNA-C* was released to replace another DNA-L* from DNA-C-L*. In this cycle, a lot of free DNA-L* could be obtained. Subsequently, free DNA-L* entered cycle II which was also induced by Exo III. In cycle II, AuNPs modified with DNA-L (AuNPs-L) were hired to produce color signal. Free DNA-L* could bind with its complementary chain DNA-L to form AuNPs-L-L* duplex structure. This structure could also be cut by Exo III. After the AuNPs-L-L* duplex was degraded, DNA-L* was released again and induced more DNA-L to be degraded. As a result, AuNPs-L became AuNPs. Finally, the AuNPs without DNA protection could aggregate in a high-salt solution, which produced a color change from red to violet (Figure 3a). With two rounds of the signal amplification (cycle I and II), the colorimetric and visual Hg^2+^ biosensor was realized with a detection limit as low as 0.9 nM and an extra wide detection range of 1 nM to 10 μM.

In 2018, Song et al. reported a ultrasensitive electrochemical for the detection Hg^2+^ based on Exo III-assisted double-cycle signal amplification strategy and gold electrodes (AuE) [51]. In this DNA biosensor, a label-free hairpin probe (HP1) was designed which had a protruding 3′-terminal. The 3′-terminal contained a lot of T bases for targeting Hg^2+^. Another hairpin probe (HP2) was modified on the surface of the AuE by its 5′-terminal. The 3′-terminal of HP2 was labeled by methylene blue (MB). In the absence of Hg^2+^, the hairpin structure of HP2 was not open. MB and AuE were close enough to induce a large electrical signal. In the presence of Hg^2+^, the primer DNA could bind HP1 to form the duplex structure via T-Hg-T mismatch. This structure could then be digested from the 3′-terminal of HP1. As a result, the 5′-terminal of HP1 (Helper), primer DNA and Hg^2+^ were all released. The primer DNA and Hg^2+^ could bind another HP1 to form another new Helper. Thus, more Helper could be obtained in cycle I. In cycle II, Helper could bind HP2 to open the hairpin structure by forming another duplex structure (Helper-HP2). The Helper-HP2 duplex could be recognized by Exo III. Along with the procedure that Exo III digested Helper-HP2 from the 3′-terminal of HP2, MB and Helper were released. Free Helper could bind another HP2 to release more free MB (Figure 3b). As a result, free MB was farther from AuE, thus the electrical signal had a distinct decrease. The detection limit of this ultrasensitive electrochemical biosensor was 227 pM.

In 2020, Zhou et al. designed a fluorescence DNA biosensor for the Hg^2+^ detection using a similar signal amplification strategy and G-quadruplex/NMM as a reporter [52]. In this DNA biosensor, two hairpins DNA (hairpin A and hairpin B) were designed with 3′-protruding terminals which could resist the Exo III digestion. In the presence of Hg^2+^, Hg^2+^ could help DNA1 hybridize with the 3′-terminal of hairpin A via T-Hg-T mismatch. The DNA1- hairpin A could be cut by Exo III form the 3′-terminal of hairpin A. Then, DNA1, Hg^2+^ and the 5′-terminal of hairpin A (e-d-c) were released. Afterwards, DNA1, Hg^2+^ could activate another round of the cleavage reaction to produce more e-d-c. Likewise, the e-d-c hybridized with the 3′-terminal of Hairpin B via T-Hg-T mismatch. Under the Exo III digestion, the 5′-terminal of hairpin B was obtained which was also DNA e-d-c. DNA e-d-c was released and reused to trigger the next round digestion (Figure 3c). At last, large amounts of DNA e-d-c were obtained through two rounds of signal amplification (cycle I and II). Then, DNA e-d-c with G-rich sequence could form G-quadruplex structure in the presence of K^+^. Finally, G-quadruplex structure could bind with NMM to emit intense fluorescence. The autocatalytic DNA biosensor was very sensitivity with the detection limit of 10 fM.

The Exo III-assisted signal amplification strategy can also be used in combination with other amplification methods [53]. In 2021, He et al. reported a triple signal amplification strategy for ultrasensitive Hg^2+^ detection based on a DNA dual cycles, organic-inorganic hybrid nanoflowers (Cu_3_(PO_4_)_2_HNFs) and AuNP probe (Figure 3d) [54]. In the first strategy, hairpins DNA (HP1) containing report DNA (RDNA) was designed. In the presence of Hg^2+^, it caused the two ends of HP1 to form double-stranded structures via T-Hg-T mismatch. The cycle was triggered by Exo III to digest the 3′-end of HP1. After the digestion, Hg^2+^ was released for the next cycle and large amounts of RDNA were obtained. The free RDNA could form a new double stranded DNA with another hairpin DNA (HP2) which had been modified on the electrode (Au/GCE). Subsequently, the third hairpin DNA (HP3) was used to open the new dsDNA. The RDNA was released again for the next cycle. At a result, HP3 was connected on the electrode by the DNA dual cycles amplification. In the second strategy, the (Cu_3_(PO_4_)_2_HNFs)@AuNPs@c-probe was synthesized. This complex was effectively anchored on the Au/GCE by complementary base between c-probe and HP3. Due to the nanoflowers with a high surface area, it could carry lots of signal molecules to amplify signal. In this paper, signal molecule R-probe@AuNPs was synthesized by modifying r-probe 1 and r-probe 2 marked with ferrocene on the AuNPs. Then, the R-probe@AuNPs was linked into the nanoflowers by c-probe. Since the AuNPs also had high surface area, it could carry many ferrocene molecules. Thus, the AuNPs acted as the third amplification strategy. Finally, this ultrasensitive electrochemical biosensor had an ultra-low detection limit of 0.19 fM by the triple signal amplification strategy.

### 2.2. Exonuclease I

Exonuclease I (Exo I) is another member of the exonuclease family. It is different from Exo III and its substrate is single-stranded DNA. The cutting directions of them are consistent, both from the 3’-end to the 5’-end. Hg^2+^ is commonly used to help form double-strands via T-Hg-T mismatch. And the double-strands cannot be digested by Exo I. However, the remaining single-stranded DNA without binding to Hg^2+^ can be cut. Thus, Exo I can also be used to design Hg^2+^ DNA biosensor. Since Hg^2+^ cannot be released repeatedly, this method is rarely used. Here, we give two examples to illustrate this method.

**Figure 4:**
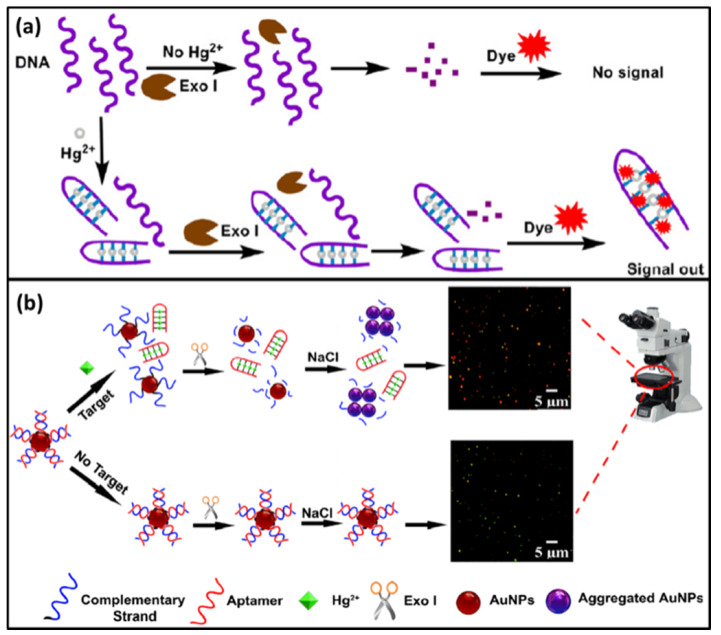
(**a**) A scheme of a label-free fluorescence biosensor for detection of Hg^2+^ based on Exo I Reprinted with permission from [55]. Copyright 2012 Elsevier. (**b**) A scheme of an optical ultrasensitive DNA biosensor to detect Hg^2+^ based on dark-field microscope and Exo I. Reprinted with permission from [56]. Copyright 2019 Springer Nature.

Yuan et al. reported a label-free fluorescence biosensor for the detection of Hg^2+^ based on Exo I [55]. In the absence of Hg^2+^, the single-stranded T-rich DNA (ssDNA) could be digested by Exo I. When the DNA dye was added, no signal was observed. In the presence of Hg^2+^, the ssDNA were folded into hairpin-like double-stranded DNAs (dsDNA). This dsDNA could not be digested by Exo I (Figure 4a). Thus, the DNA protected by Hg^2+^ could still bind with DNA dye to emit fluorescence signal. This DNA biosensor had a high sensitivity for Hg^2+^ detection with the detection limit of 15 nM.

To improve the sensitivity, some simple devices can be used in combination with Exo I. For example, Li; et al. developed an optical ultrasensitive DNA biosensor to detect Hg^2+^ by using dark-field microscope and Exo I [56]. In this biosensor, the complementary strand of Hg^2+^ aptamer was modified on the AuNPs. In the absence of Hg^2+^, the complementary strand and aptamer hybridized into a double-stranded structure (dsDNA) which could not be cut by Exo I. The dsDNA could prevent AuNPs from aggregation in the solution of NaCl. In the presence of Hg^2+^, the aptamer dissociated from the AuNPs and subsequently formed stronger interactions with T-Hg-T. Then, the single complementary strand was digested by Exo I to cause the release of AuNPs. In the solution of NaCl, the AuNPs aggregated with the color change (Figure 4b). The intensity of color change could then be observed and calculated by dark-field microscope. Because the sensitivity of the microscope was much higher than that of the naked eye, the ultrasensitive DNA biosensor was realized with the limit of detection as low as 36 fM and a linear range from 83 fM to 8.3 μM.

### 2.3. Nicking Endonuclease

Nicking endonucleases (nickases) are members of the endonuclease family. They can specifically recognize the restriction sites in the double-chain state and cut single-stranded DNA at that site. Because they only cut one strand of DNA, the other strand of DNA can be retained for signal amplification. This nickase-assisted amplification strategy has been developed for the detection of different targets including Hg^2+^ [57,58,59,60]. Depending on their recognition sequence, there are many different types of nickase such as Nt.AlWI [61], Nt.BstNBI [62], Nb.BbvC [63] and so on. The abundance of these nickases provides convenience for the design of DNA biosensor.

**Figure 5:**
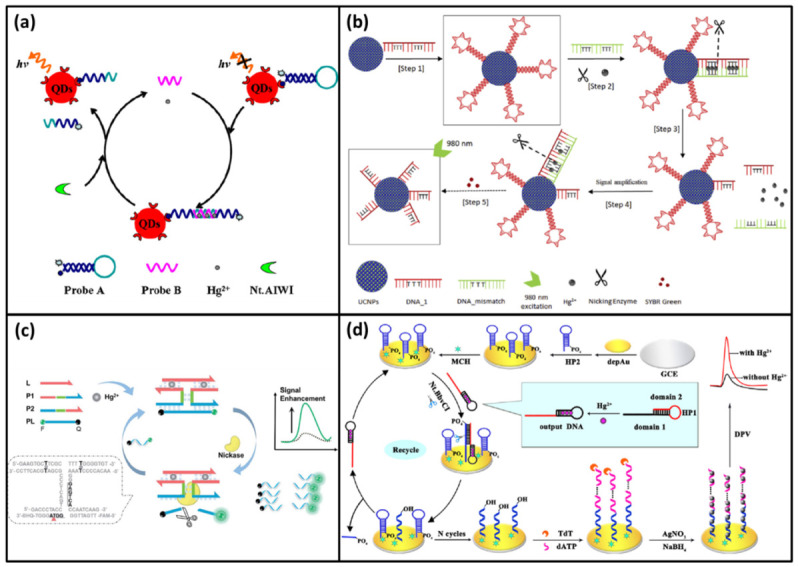
(**a**) A scheme of a nicking endonuclease assisted signal amplification for Hg^2+^ detection based on quantum dots. Reprinted with permission from ref. [65]. Copyright 2013 Elsevier. (**b**) A scheme of a highly sensitive biosensor for Hg^2+^ detection based on luminescence resonance energy transfer (LRET) between nanoparticles and SYBR Green I. Reprinted with permission from ref. [66]. Copyright 2019 Elsevier. (**c**) A scheme of an Hg^2+^ DNA biosensor based on nickase-assisted signal amplification and DNA assembly. Reprinted with permission from ref. [67]. Copyright 2019 Royal Society of Chemistry (**d**) A scheme of a sensitive electrochemical biosensor to detect Hg^2+^ based on nickase Nt.BbvCI and terminal deoxynucleotidyl transferase (TdT). Reprinted with permission from ref. [68]. Copyright 2016 Elsevier.

For example, back to 2011, Nt.AlWI was used to detect mercury ions by Li et al. [64]. In this biosensor, ferrocene (Fc)-ssDNA was modified on the Au electrode. In the presence of Hg^2+^, the Fc-ssDNA switched into a hairpin structure via T-Hg-T mismatch. The hairpin structure contained a cleavage site of Nt.AlWI. Along with the digestion of Nt.AlwI, Fc was released to increase the distance from the electrode. Thus, the electrical signal gradually decreased as the mercury ions increased. Since this method did not use cyclic amplification, the limit of detection was at the micromole level. Subsequently, Ma et al. reported a signal amplification biosensor that also used Nt.AlwI [65]. In this DNA biosensor, the streptavidin-coated quantum dots (QDs) was used to conjugate biotinylated hairpin-shaped probe A with a quencher. The probe A had a cleavage site of Nt.AlwI in the loop region. Due to the single state, it could not be cut by Nt.AlwI. At the beginning, the fluorescence of the QDs was quenched by quencher due to the short distance. In the presence of Hg^2+^, another DNA probe B hybridized with probe A to form a duplex structure via T-Hg-T mismatch. Probe A in the duplex structure could be digested to increase the distance between the quencher and the QDs. Thus, the fluorescence of the QDs was recovered. Meanwhile, the intact probe B and Hg^2+^ entered a new cycle to digest more probe A (Figure 5a). After the cyclic amplification, a remarkable signal was achieved. With the help of Nt.AlwI, the sensitivity of this biosensor was increased with the detection limit of 0.8 nM.

In recent years, nickase-assisted amplification strategy has also been reported for more DNA biosensor to detection of Hg^2+^. In 2019, Vijayan et al. developed a highly sensitive biosensor for Hg^2+^ detection based on luminescence resonance energy transfer (LRET) between nanoparticles and SYBR Green I [66]. In this biosensor, the upconversion nanoparticles NaYF_4_: Yb^3+^/Tm^3+^ (UCNPs) were synthesized and modified by hairpin DNA (DAN-1) containing thymine bases. At the beginning, DAN-1 could form a hairpin structure to bind SYBR Green I. Under the excitation at 980 nm, the UCNPs had strong visible bands at 477, 650 and 800 nm. And the absorption of the SYBR Green I was exactly 477 nm. Thus, the emission intensity of 477 nm decreased. In the presence of Hg^2+^, DAN-1 bound with DNA- mismatch to form a completely complementary dsDNA via T-Hg-T mismatch. This structure could be recognized by nicking enzyme. Then, DAN-1 was digested; the DNA- mismatch and Hg^2+^ were released to trigger a new cycle (Figure 5b). Since DNA-1 was degraded, SYBR Green I was also free. Therefore, the 477 nm emission of UCNPs was recovered. With the help of nickase-assisted amplification strategy, the sensitivity of this biosensor was as low as 0.14 nM.

In 2019, Liu et al. designed an Hg^2+^ DNA biosensor based on nickase-assisted signal amplification and DNA assembly [68]. In this biosensor, another nickase, Nt.BstNBI, was employed. Besides, four DNA probes were designed. They were a recognition probe (L), semi-enzyme-A (P1), semi-enzyme-B (P2) and fluorescein/quencher labeled reporting probes (PL), respectively. Without Hg^2+^, the four probes were all free. No fluorescence signal was observed, because of the intact quenching system. When Hg^2+^ was added, L, P1 and P2 could bind together to form an I-shaped DNA assembly structure via T-Hg-T mismatch (Figure 5c). Then PL could hybridize with this I-shaped structure by base pairing. Then, the cleavage sites of Nt.BstNBI appeared in the stem of the I-shaped junction. With Nt.BstNBI added, PL was cut to separate fluorescein from the quencher. Thus, the fluorescence signal could be recorded. Meanwhile, the I-shaped structure was also released to hybridize with another PL. At last, a lot of PL was cleaved to emit strong fluorescent signal in the presence of a small amount of Hg^2+^. The detection limit of this simple biosensor was 1.7 nM.

In addition, nickase can also be used in combination with other enzymes to improve the sensitivity of DNA biosensor. In 2016, the Yuan group developed a sensitive electrochemical biosensor to detect Hg^2+^ based on nickase Nt.BbvCI and terminal deoxynucleotidyl transferase (TdT) [67]. In this biosensor, two hairpins DNA (HP1 and HP2) were designed. HP1 was a T-rich DNA. Without Hg^2+^, its 5′-end was covered by forming hairpin structure. In the presence of Hg^2+^, the 5′-end exposed to form output DNA by conformation changes via T-Hg-T mismatch. HP2 was modified on the Au/GCE electrode by its 5′-end. The 3′-end was labeled by PO_4_ to block the TdT reaction. The output DNA could hybridize with HP2 to open the hairpin structure and form the output DNA-HP2 complex. This complex could be recognized by Nt. BbvCI and the 3′-PO_4_ of HP2 was cut off. Meanwhile, the output DNA was released to hybridize with another HP2 (Figure 5d). Accompanying with the cleaving of HP2, the substantial 3′-OH group exposed. Then, 3′-OH of HP2 could be added with a lot of deoxyadenosine triphosphate (dATP) to form a long ssDNA nanotail with the help of TdT and dATP. The ssDNA nanotail with negatively charged DNA skeleton could absorb the positively charged silver ions. In the presence of reduction regent sodium borohydride, the silver ions transformed into silver nanoparticles. The nanoparticles could deposit on electrode surface to output electrochemical signal. With the help of two enzymes, the sensitivity of this biosensor was improved with an ultralow detection limit of 3 pM.

### 2.4. Duplex-Specific Nuclease

**Figure 6:**
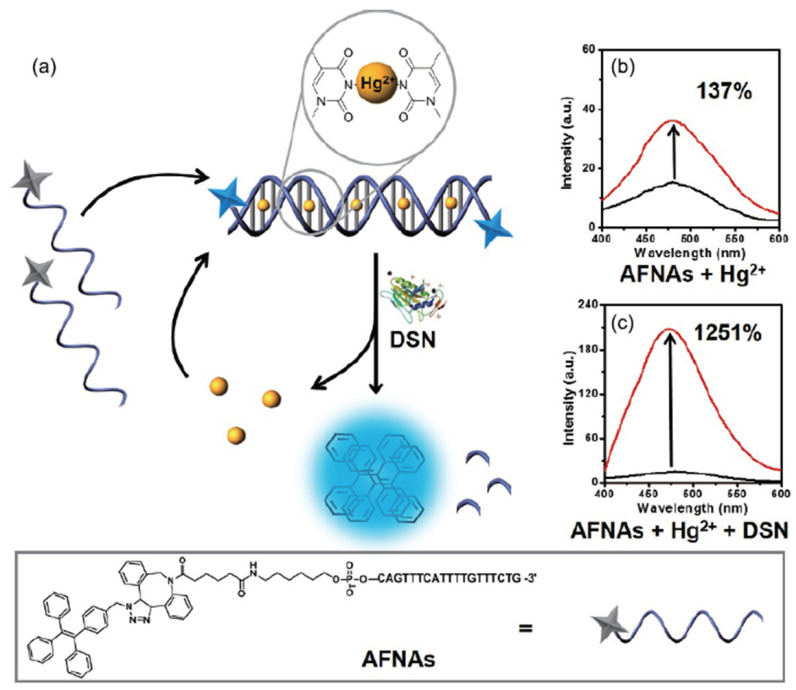
A scheme of a highly sensitive fluorescence biosensor to test Hg^2+^ based on DSN and AIE. Reprinted with permission from [69]. Copyright 2017 Springer Nature.

Duplex-specific nuclease (DSN) is another member of the endonuclease family. It can selectively cleave dsDNA or the DNA in DNA-RNA hybrid duplexes. However, it cannot cut the ssDNA. Since Hg^2+^ can help ssDNA fold into dsDNA which can be digested by DSN to release Hg^2+^, DSN can also be used to amplify signal. In 2017, Xia group designed a highly sensitive fluorescence biosensor to test Hg^2+^ based on DSN and aggregation-induced emission (AIE) [69]. In this biosensor, fluorogen TPE was modified on the ssDNA to form a simple AIEgens functional nucleic acids (AFNAs) probe. In the absence of Hg^2+^, AFNAs probe exhibited a weak fluorescence signal. In the presence of Hg^2+^, the AFNAs probe changed the conformation from ssDNA to dsDNA via T-Hg-T mismatch. Then, the dsDNA was digested by DSN to released Hg^2+^ and fluorogen TPE. Hg^2+^ could be reused to bind new AFNAs probe. The fluorogen TPE could form supramolecular aggregate to enhance the fluorescence signal by AIE phenomenon (Figure 6). The sensitivity of this DNA biosensor was improved because Hg^2+^ was reused with the help of DSN. The detection limit of this simple DNA biosensor was 10 fM with a good linearity range from 10 fM to 1 nM.

### 2.5. Deoxyribonuclease I

Deoxyribonuclease I (DNase I) is DNA specific endonuclease which can degrade dsDNA and ssDNA. Mechanically, ssDNA can be absorb on the nanomaterial, while Hg^2+^ can shed DNA from the nanomaterial by promoting ssDNA to dsDNA via T-Hg-T mismatch. Then, free dsDNA can be digested by DNase I to release Hg^2+^. Thus, DNase I also can be used to amplify signal.

**Figure 7:**
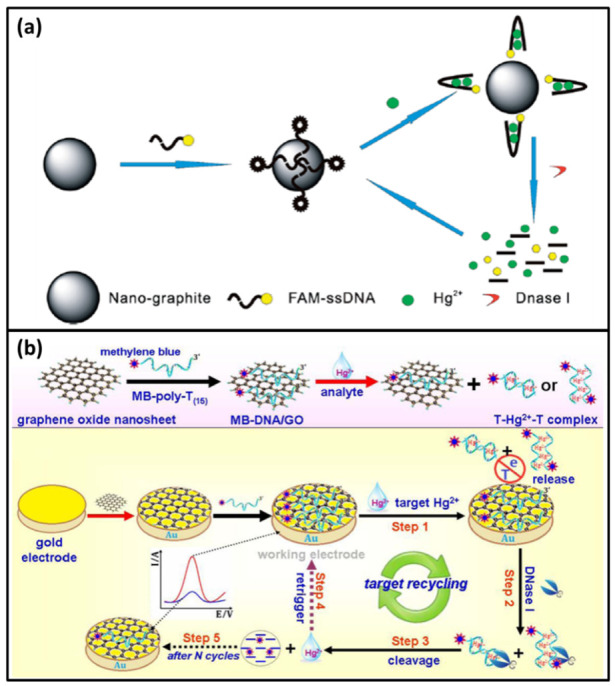
(**a**) A scheme of a nano-graphite-DNA hybrid fluorescent biosensor for Hg^2+^ detection based on DNase I. Reprinted with permission from [70]. Copyright 2014 Royal Society of Chemistry (**b**) A scheme of a novel electrochemical biosensor to monitor Hg^2+^ based on DNase I-assisted target recycling. Reprinted with permission from [73]. Copyright 2016 Elsevier.

Wei et al. designed a nano-graphite-DNA hybrid fluorescent biosensor for Hg^2+^ detection based on DNase I for the first time [70]. In this biosensor, a T-rich ssDNA was labeled by fluorophore FAM. Without Hg^2+^, ssDNA was absorbed on the nano-graphite by π-π stacking [71,72] and the fluorescence of FAM was quenched by nano-graphite. Thus, no fluorescence signal was obtained. In the presence of Hg^2+^, ssDNA folded into dsDNA via T-Hg-T mismatch to detach from the nano-graphite surface. Free ssDNA could be cleaved by DNase I to release Hg^2+^ and FAM. The fluorescence of FAM was recovered. Hg^2+^ was reused in next cycle to lead to the signal amplification (Figure 7a). This amplified biosensor was simple and cost-effective with a detection limit of 0.5 nM.

Recently, Lu et al. reported a novel electrochemical biosensor to monitor Hg^2+^ based on DNase I-assisted target recycling [73]. In this biosensor, a methylene blue (MB)-labeled poly-T_(15)_ oligonucleotide probe was designed to generate electrochemical signal. At the beginning, the probe was attached on the graphene oxide nanosheets/Au electrode by π-π stacking [74]. Since the graphene oxide nanosheets could protect MB-poly-T_(15)_ from DNase I cleavage. The electrochemical signal was high. When Hg^2+^ was added, the MB-poly-T_(15)_ ssDNA folded into dsDNA via T-Hg-T mismatch. And dsDNA was released from the electrode. Then, it was cut by DNase I to release free Hg^2+^ and MB. Free Hg^2+^ entered the next cycle to release numerous MB (Figure 7b). Due to the distance increasing between MB and the electrode, the electrical signal decreased. By the DNase I-triggered target recycling reaction, the biosensor had a high sensitivity with a detection limit of 0.12 nM.

## 3. DNAzyme

**Figure 8:**
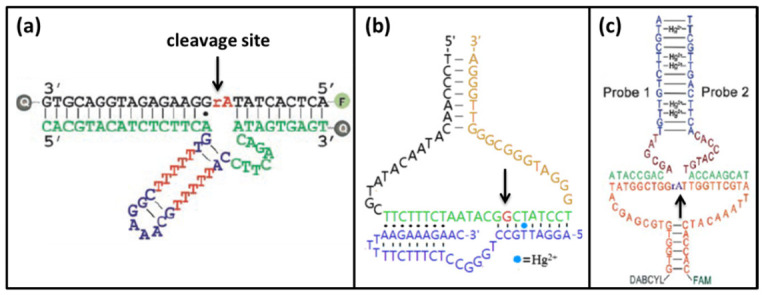
The structures and functions of modified DNAzyme for Hg^2+^ detection. (**a**) modified UO_2_^2+^-ion-specific DNAzyme; Reprinted with permission from [18]. Copyright 2007 Wiley VCH. (**b**) Cu^2+^-specific DNAzyme; Reprinted with permission from [85]. Copyright 2012 Elsevier. (**c**) Mg^2+^-specific DNAzyme. Reprinted with permission from [86]. Copyright 2012 Royal Society of Chemistry.

DNAzyme is a kind of DNA with catalytic activity. Its chemical nature is deoxyribonucleotides which is different from nuclease. The ribozyme (RNAzyme or DNAzyme) was discovered earlier than protease. The first RNAzyme was the ribosomal RNA intervening sequence of Tetrahymena which was discovered in 1982 [75], the first DNAzyme named GR5 was isolated by Breaker and Joyce in 1994 [76]. These discoveries changed the notion of enzyme that all enzymes were proteins. Many DNAzymes require metal ions as cofactors to attack phosphorus atoms in the substrate phosphoric acid skeleton, and many DNAzymes were screened in vitro [77,78,79]. Thus, the DNAzymes can at least be used to design a biosensor to detect their cofactors. Because Hg^2+^ has a weak interaction with phosphate, and high concentration of Hg^2+^ can cause the denaturation of DNA, it is difficult to obtain Hg^2+^-specific DNAzyme [80]. The Hg^2+^-specific DNAzyme named 10–13 was selected using amino/imidazole modified library by Perrin group, and then this group designed a DNAzyme biosensor for Hg^2+^ [81]. The base modifications in 10-13 DNAzyme limited its application in biosensor design. Thus, few biosensors had been reported based on Hg^2+^-specific DNAzyme. Most DNAzyme biosensors for Hg^2+^ are based on other modified DNAzymes such as UO_2_^2+^-ion-specific DNAzyme, Cu^2+^-specific DNAzyme, and Mg^2+^-specific DNAzyme (Figure 8) [31,82,83,84].

### 3.1. UO_2_^2+^-Specific DNAzyme

**Figure 9:**
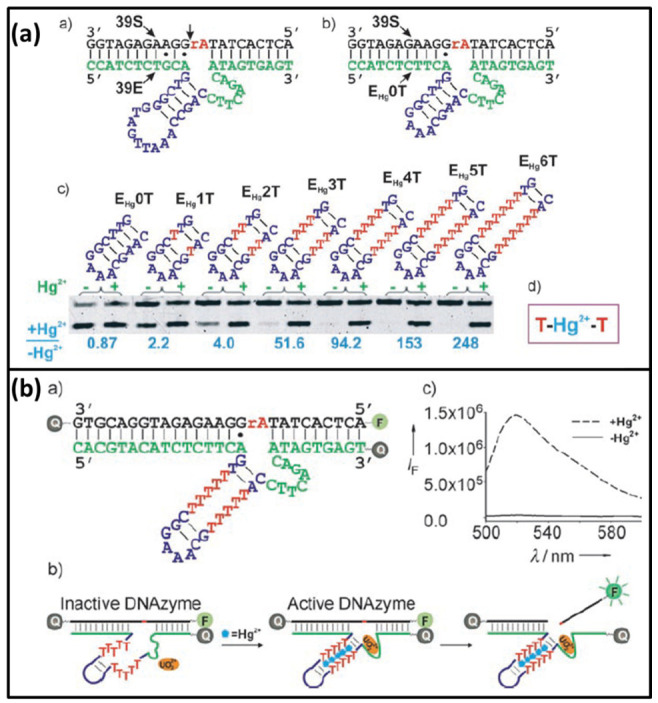
(**a**) The secondary structure of the originally UO_2_^2+^-ion-specific DNAzyme, the new DNAzyme with the replaced stem loop, and the stem-loop part of new DNAzymes with 0-6 T-T mismatches. (**b**) The secondary structure of the Hg^2+^ biosensor DNAzyme and the scheme of biosensor design. Reprinted with permission from [18]. Copyright 2007 Wiley VCH.

The Lu group designed an ultrahigh sensitivity and selectivity Hg^2+^ biosensor based on modified UO_2_^2+^-ion-specific DNAzyme [18]. The originally UO_2_^2+^-ion-specific DNAzyme contains a substrate strand (39S) and an enzyme strand (39E) which contains an eight-nucleotide bulge and a stem loop [87]. This group found that changing the base of the stem loop had no effect on the activity and the role of stem loop was only structural maintenance. When the stem loop was replaced and one of the A·G mismatches on the left side of cleavage site was replaced by an A-T base pair to form E_Hg_0T, the new DNAzyme was still active in the presence of UO_2_^2+^-ion. Then, other mutant DNAzyme with different amounts of T-T mismatches in the stem region was tested to observe the enzyme activity. Because E_Hg_5T had no activity in the absence of Hg^2+^, E_Hg_5T was chosen to design Hg^2+^ biosensor (Figure 9a). At the beginning, the substrate 39S was labeled by the fluorophore and quencher. At one end close to the fluorophore, the new DNAzyme was also modified by the quencher to further quench the fluorescence. Thus, no fluorescence signal was observed without Hg^2+^. In the presence of Hg^2+^, Hg^2+^ folded the new DNAzyme into an active conformation. With UO_2_^2+^-ion, the new DNAzyme could cleave its substrate to release fluorescence (Figure 9b). Since the new DNAzyme was not damaged, it could enter the next cycle to bind with another substrate, releasing more fluorescence. The detection limit of this biosensor was 2.4 nM.

### 3.2. Cu^2+^-Specific DNAzyme

**Figure 10:**
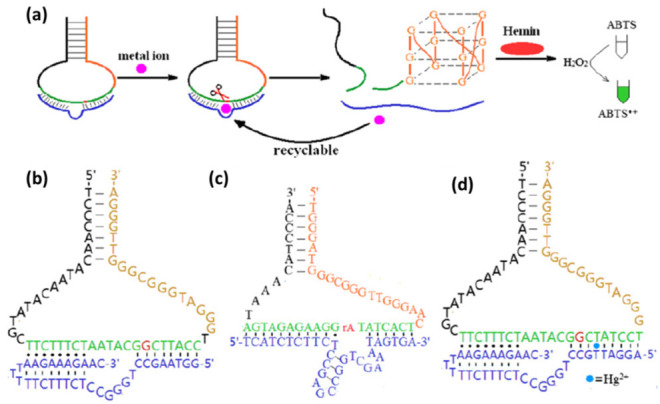
(**a**) The scheme of DNA biosensor for detecting metal ion including Hg^2+^ based on the Cu^2+^-specific DNAzyme and G-quadruplex DNAzymes. (**b**) The Cu^2+^-specific DNAzyme, (**c**) Pb^2+^-specific DNAzyme, and (**d**) the modified Cu^2+^-specific DNAzyme for Hg^2+^ biosensor. Reprinted with permission from [85]. Copyright 2012 Elsevier.

Zhang et al. developed an Hg^2+^ biosensor based on the Cu^2+^-specific DNAzyme and G-quadruplex DNAzymes [85]. Similar to the UO_2_^2+^-ion-specific DNAzyme, they engineered the Cu^2+^-specific DNAzyme to recognize Hg^2+^. The originally Cu^2+^-specific DNAzyme was shown in Figure 10b. The T-A Watson-Crick base pair in the 5′-end of DNAzyme was replaced by a T·T mismatches to construct modified new DNAzyme (Figure 10d). Without Hg^2+^, the new DNAzyme could not associate with the substrate strand to form a stable enzyme/substrate complex. Thus, the substrate was free from being cleaved. The design strategy of this biosensor was shown in Figure 10a. A hairpin DNA contained a G-rich sequence and a substrate sequence was used in this biosensor. At the beginning, the G-rich sequence was covered by forming hairpin structure. Thus, the G-quadruplex DNAzyme could not be formed to oxidize ABTS color reaction. In the presence of Hg^2+^, the new DNAzyme could bind the substrate via T-Hg-T mismatch. Then, the hairpin substrate was cut in the presence of Cu^2+^. As a result, the G-rich sequence was released to form G-quadruplex DNAzymes which improved the oxidation activity of Hemin [88,89,90]. Then, ABTS was oxidized to produce the color. Meanwhile, the new DNAzyme entered the next cycle to split another hairpin substrate. The sensitivity of this biosensor was improved by DNA/RNA-cleaving DNAzyme and the G-quadruplex DNAzyme. Therefore, this biosensor had a high level of sensitivity with the detection limit of 4 nM.

### 3.3. Mg^2+^-Specific DNAzyme

**Figure 11:**
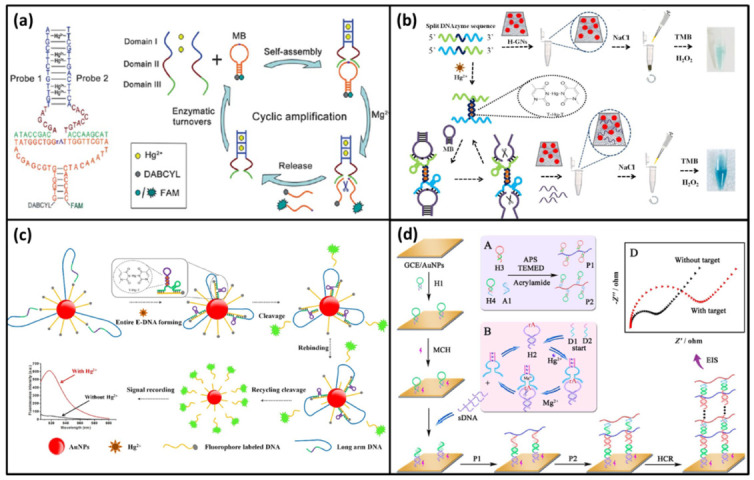
(**a**) The scheme of a fluorescence DNA biosensor to detect Hg^2+^ based on splitting Mg^2+^-dependent DNAzyme. Reprinted with permission from [86]. Copyright 2012 Royal Society of Chemistry. (**b**) The scheme of a visual and sensitive DNA biosensor for Hg^2+^ detection based on split DNAzyme amplification and peroxidase-like activity of hemin-graphene composites (H-GNs). Reprinted with permission from [92]. Copyright 2019 Elsevier. (**c**) The scheme of a simple and amplified Hg^2+^ detection strategy based on DNAzyme motor. Reprinted with permission from [93]. Copyright 2018 Elsevier. (**d**) The scheme of an electrochemical impedance biosensor for Hg^2+^ detection based on DNAzyme-assisted target recycling and hybridization chain reaction (HCR). Reprinted with permission from [94]. Copyright 2017 Elsevier.

As early as 1995, Breaker and Joyce obtained the Mg^2+^-dependent DNAzyme by in vitro selection [91]. This DNAzyme has high catalytic activity. Meanwhile, Mg^2+^ is a very common ion and is not harmful to the environment. Thus, modified Mg^2+^-dependent DNAzyme is well adapted to detect mercury ions. Qi, et al. designed a fluorescence DNA biosensor to detect Hg^2+^ based on split Mg^2+^-dependent DNAzyme [86]. In this biosensor, the Mg^2+^-dependent DNAzyme was artificially split into two separate fragments (probe1 and probe2). The T-rich sequence was added at the 5′-end of probe1 and the 3′-end of probe2. Meanwhile, the hairpin structure substrate with fluorescence and quencher was designed (Figure 11a). In the absence of Hg^2+^, the split Mg^2+^-dependent DNAzyme had no activity, and the substrate could not be cut. Thus, no fluorescent signal emitted. In the presence of Hg^2+^, probe1 and probe2 could combine to form a complete DNAzyme via T-Hg-T mismatch. With Mg^2+^, the hairpin substrate was cleaved to cause the separation of the fluorophore and the quencher. Therefore, the fluorescent signal could be recorded. After that probe1 and probe2 was released, the DNAzyme entered the next cycle to cleave another substrate. Then, stronger fluorescence could be observed. The detection limit of this amplified fluorescence biosensor was down to 0.2 nM.

In 2018, Deng et al. developed a visual and sensitive DNA biosensor for Hg^2+^ detection based on split DNAzyme amplification and peroxidase-like activity of hemin-graphene composites (H-GNs) [92]. In this biosensor, two split DNAzyme sequences were designed which contained partly E-DNA sequences at both ends and thymine bases in the middle. With the help of Hg^2+^, two entire E-DNA could be formed via T-Hg-T mismatch. Then, the entire E-DNA with high activity could cleave its molecular beacon (MB) substrate to result DNA fragments in the presence of Mg^2+^. When the DNA fragments were released, the E-DNA can hybridize with another MB substrate to cause more DNA fragments. The large amount of DNA fragments adsorbed on the surface of H-GNs to prevent forming aggregation. Then, TMB could be oxidized to produce color (Figure 11b). Without Hg^2+^, the split DNAzyme could not cleave MB substrate. Without the protection of DNA fragments, H-GNs would aggregate to lose peroxidase-like activity. Then, no color reaction happened. This visual and DNAzyme amplified biosensor had a low detection limit of 33 pM.

In 2018, Yun et al. reported a simple and amplified Hg^2+^ detection strategy based on DNAzyme motor [93]. In this biosensor, fluorophore labeled DNA probes and long arm DNA were modified on the surface of AuNPs via Au-S bond. The fluorophore-labeled DNA probes contained the substrate of Mg^2+^-specific DNAzyme. At the initial status, the fluorescent signal was quenched. The long arm DNA had a split E-DNA which was separated by T-rich sequence. In the absence of Hg^2+^, the split E-DNA had no activity and the fluorophore-labeled substrate could not be cleaved. Thus, no fluorescent signal could be observed. When Hg^2+^ was added, the split E-DNA was able to form an entire E-DNA via T-Hg-T interaction. Then the substrate was cleaved to release fluorophore in the presence of Mg^2+^. The free entire E-DNA could hybridize with another substrate to trigger the next cycle (Figure 11c). At last, more fluorophore molecules were released to bring significant fluorescent signal. The limit of detection of this biosensor was 30 pM.

Cai; et al. developed an electrochemical impedance biosensor for Hg^2+^ detection based on DNAzyme-assisted target recycling and hybridization chain reaction (HCR) [94]. In this biosensor, four hairpins DNA (H1, H2, H3, and H4) and a split Mg^2+^-specific DNAzyme were designed. The capture probe H1 was modified on the surface of GCE/AuNPs electrode. H2 contained the substrate sequence of DNAzyme. H3 and H4 were synthesized from two different copolymer chains P1 and P2 by acrylamide polymer, respectively. In the presence of Hg^2+^, the split DNAzyme could form an active entire DNAzyme. This entire DNAzyme could cleave H2 to release the partial substrate strand (sDNA) in the presence of Mg^2+^. Similar to the method above, DNAzyme was reused to cleave H2 thus to obtain more sDNA. Subsequently, sDNA could hybridize with H1 to open the hairpin structure. As a result, the terminal of H1 exposed, which could hybridize with P1 to open the hairpin structure of P1. Then, the terminal of P1 further hybridized with P2 to open the hairpin structure of P2. The HCR between P1 and P2 was initiated to assemble a nonconductive DNA hydrogel film on the surface of the electrode (Figure 11d). Thus, the electronic transfer was greatly hindered. By measuring the impedance signal, the ultrasensitive biosensor of Hg^2+^ was obtained with a low detection limit of 0.042 pM.

## 4. Applications of Hg^2+^ Biosensors in Real Samples

With good detection mechanism, these Hg^2+^ DNA biosensors were also applied in real samples to evaluate the reliability and applicability. Most of these biosensors performed high selectivity in the detection process due to the specificity of T-Hg-T mismatch (Table 2). A colorimetric biosensor based on Exo III-assisted target recycling with one cycle and gold nanoparticles was used to detect Hg^2+^ in tap water samples and lake water samples by Hong et al. [46]. By determining the recovery of spiked Hg^2+^, the values of the recovery were between 92% and 106%. Meanwhile, the results were identical to those obtained by ICP-MS. The above data indicated that this biosensor had favorable potential to be applied in real samples. The electrochemical based on Exo III-assisted target recycling with two cycles was applied to detect Hg^2+^ in the tap water by Song et al. [51]. Three water samples were prepared by adding different concentrations of Hg^2+^ (10, 100, 1000 nM). Then the concentration of Hg^2+^ was measured by this biosensor with the linear range of 500 pM–5 μM and the standard atomic fluorescence spectroscopy (AFS). The results showed that this biosensor was in good agreement with AFS and the discrepancies between the two methods were all less than 14.2%. These results revealed that this biosensor might hold great potential for real sample detection. Liu et al. designed a biosensor based on nickase-assisted signal amplification to detect Hg^2+^ in in the tap water [68]. Initially, the tap was wrapped with a 0.22 mm membrane to filter the water. Then, the tap water was spiked with different concentrations of Hg^2+^. Finally, Hg^2+^ was quantitatively detected by the linear equation of the proposed biosensor. The recovery values were in the range of 94.2–111.4%, and the relative standard deviation (RSD) was between 3.8–5.0%. These results showed that this biosensor was satisfactory in monitoring the tap water. Qi developed a biosensor based on Mg^2+^-specific DNAzyme to test Hg^2+^ in Xi’an Chan River containing a variety of interference [86]. At the beginning, the river water samples were simply filtered to remove the insoluble substances. Then, the spiked water samples were prepared with different concentrations of Hg^2+^. At last, the level of Hg^2+^ was measured. The recoveries of this biosensor were between 96% and 105%. The results indicated that no obvious Hg^2+^ contamination was found in the Xi’an Chan River and the proposed biosensor could accurately measure Hg^2+^ in real samples.

## 5. Summary and Future Perspectives

In this article, we reviewed the enzyme-driven signal amplification strategy in the construction of DNA biosensors for Hg^2+^ detection. The enzymes were divided into nucleases and DNAzymes according to their chemical nature. The component of nucleases is protein, while DNAzymes consist of nucleic acids. By contrast, DNA is more stable, cheaper, and easier to modify than protein. However, a wide variety of nucleases are available for designing Hg^2+^ biosensor including Exo III, Exo I, Nickase, DSN, and DNase I. In the nucleases section, the Exo III-driven signal amplification strategy was introduced in detail. Since Hg^2+^ can help ssDNA fold into dsDNA via T-Hg-T, and the substrate of Exo III is dsDNA, Exo III can be used to design Hg^2+^ biosensor flexibly. In the DNAzymes section, it is difficult to obtain Hg^2+^-specific DNAzyme. Most of the DNAzymes used in Hg^2+^ biosensor are modified from DNAzymes which are specific for other metal ions, for example, the Mg^2+^-specific DNAzyme. These approaches with the help of other metal ion-specific DNAzyme provide a new way to design biosensors. The biosensors based on different enzymes were compared in Table 2. In ion specificity, they were all highly selective with the same recognition mechanism based on T-Hg-T mismatch. In sensitivity, the detection limit of Exo III with one cycle was range from 1 pM–380 pM. When another cycle was introduced, the detection limit can be improved to 10 fM. The highest sensitivity with the detection limit of 0.19 fM could be obtained when the nanoflowers were used in combination with Exo III. Exo I performed slightly less effective. However, when it is combined with microscope, the sensitivity can be greatly improved. The detection limit of nickase is 0.8 nM–1.7 nM. When it is combined with other enzymes such as TdTase, the sensitivity can also be remarkably improved. For example, DSN has a satisfactory sensitivity 10 fM. The sensitivity of DNase I, UO_2_^2+^-ion-specific DNAzyme and Cu^2+^-specific DNAzyme is similar to that of nickase. Moreover, the Mg^2+^-specific DNAzyme shows the same sensitivity as Exo III. The sensitivity of Mg^2+^-specific DNAzyme can also be increased with other amplification strategy such as HCR (Table 2). In summary, the sensitivity of the developed methods can fully meet the requirements of Hg^2+^ detection in drinking water (MRL 10 nM). The Exo III, DSN and Mg^2+^-specific DNAzymes have high sensitivity. The nickase, DNase I, UO_2_^2+^-ion-specific DNAzyme and Cu^2+^-specific DNAzyme have a moderate sensitivity. Therefore, when we design Hg^2+^ biosensor based on enzyme-driven signal amplification strategy in the near future, it will be better to select Exo III, DSN and Mg^2+^-specific DNAzyme as tool enzymes. Meanwhile, it is worth noting that increasing cycle, combining with nano-materials, instrument, other enzymes and other amplification strategy will improve the sensitivity of the biosensors. Thus, one future direction may focus on how to combine other ways to improve the sensitivity. The second future direction is to detect methyl mercury. Methyl mercury is another form of mercury, and this form is much more toxic which can cause permanent damage to the brain. However, the biosensors for methyl mercury have been reported much fewer [95,96,97]. This is a challenge for all the researchers in this filed. The third future direction is the simultaneous detection of multiple metal ions [98,99,100]. Since more than one toxic metal ion should be detected for ensuring people’s health, simultaneous detection of multiple ions will greatly save cost and time.

## Figures and Tables

**Table 1:** The functions of different nucleases mentioned in this review paper.

Type	Nuclease	Substrate	Degradation Direction	References
Exonuclease	Exo III	dsDNA	Form 3′ to 5′	[37,38]
Exonuclease	Exo I	ssDNA	Form 3′ to 5′	[37,38]
Endonuclease	Nickase	One stand of DNA on dsDNA	Not Applicable	[37]
Endonuclease	DSN	dsDNA	Not Applicable	[38]
Endonuclease	DNase I	ssDNA/dsDNA	Not Applicable	[37]

**Table 2:** Comparison of biosensors based on different enzymes for mercury detection

Enzyme	Detection Limit	Linear Range	Ion Specificity	Recovery in Real Samples	References
Exo III-one cycle	2.3 pM	5 pM–10 nM	High	95.7–102%	[43]
Exo III-one cycle	1 pM	1 pM–500 nM	High	Not given	[26]
Exo III-one cycle	3.2 pM	10 pM–100 nM	High	92–106%	[46]
Exo III-one cycle	380 pM	1 nM–500 nM	High	97.16–106.61%	[47]
Exo III-Two cycles	0.9 nM	1 nM–10 μM	High	91.2–105.9%	[50]
Exo III-Two cycles	227 pM	500 pM–5 μM	High	Not given	[51]
Exo III-Two cycles	10 fM	10 fM–100 nM	High	88–105%	[52]
Exo III combination with nanoflowers	0.19 fM	1 fM–10 nM	High	94.30–106.91%	[54]
Exo I	15 nM	Not given	High	Not given	[55]
Exo I combination with microscope	36 fM	83 fM–8.3 μM	High	96–104%	[56]
Nickase	0.8 nM	1 nM–15 nM	High	93.3–103.8%	[65]
Nickase	0.14 nM	0–2.0 nM	High	Not given	[66]
Nickase	1.7 nM	5 nM–250 nM	High	94.2–111.4%	[68]
Nickase combination with TdTase	3 pM	10 pM–100 nM	High	91.5–108.8%	[67]
DSN	10 fM	10 fM to 1 nM	High	97.4–106.8%	[69]
DNase I	0.5 nM	0–200 nM	High	Not given	[70]
DNase I	0.12 nM	0.5 nM–50 nM	High	Not given	[73]
UO_2_^2+^-specific DNAzyme	2.4 nM	0–20 nM	High	Not given	[18]
Cu^2+^-specific DNAzyme	4 nM	0–20 nM	Good	91.3–109.5%	[85]
Mg^2+^-specific DNAzyme	0.2 nM	1nM–20 nM	Ultrahigh	96–105%	[86]
Mg^2+^-specific DNAzyme	33 pM	50 pM–1.2 nM	Good	91.0–108.4%	[92]
Mg^2+^-specific DNAzyme	30 pM	0.1 nM–5 nM	Good	94–108%	[93]
Mg^2+^-specific DNAzyme combination with HCR	42 fM	0.1 pM–10 nM	Good	95.2–103.3%	[94]

## Data Availability

The datasets used and/or analyzed and reported in this review are available from the corresponding author on reasonable request.
